# How Thermophilic Gram-Positive Organisms Perform Extracellular Electron Transfer: Characterization of the Cell Surface Terminal Reductase OcwA

**DOI:** 10.1128/mBio.01210-19

**Published:** 2019-08-20

**Authors:** N. L. Costa, B. Hermann, V. Fourmond, M. M. Faustino, M. Teixeira, O. Einsle, C. M. Paquete, R. O. Louro

**Affiliations:** a Instituto de Tecnologia Química e Biológica António Xavier, Universidade NOVA de Lisboa, Lisbon, Portugal; b Institut für Biochemie, Albert-Ludwigs-Universität Freiburg, Freiburg im Breisgau, Germany; c Aix-Marseille Université, Centre National de la Recherche Scientifique (CNRS), Marseille, France; d BIOSS Centre for Biological Signalling Studies, Albert-Ludwigs-Universität Freiburg, Freiburg im Breisgau, Germany; California Institute of Technology

**Keywords:** bioelectrochemical systems, Gram-positive bacteria, *Thermincola*, extracellular electron transfer, multiheme cytochromes, terminal oxidoreductases

## Abstract

Extracellular electron transfer is the key process underpinning the development of bioelectrochemical systems for the production of energy or added-value compounds. Thermincola potens JR is a promising Gram-positive bacterium to be used in these systems because it is thermophilic. In this paper, we describe the structural and functional properties of the nonaheme cytochrome OcwA, which is the terminal reductase of this organism. The structure of OcwA, determined at 2.2-Å resolution, shows that the overall fold and organization of the hemes are not related to other metal reductases and instead are similar to those of multiheme cytochromes involved in the biogeochemical cycles of nitrogen and sulfur. We show that, in addition to solid electron acceptors, OcwA can also reduce soluble electron shuttles and oxyanions. These data reveal that OcwA can work as a multipurpose respiratory enzyme allowing this organism to grow in environments with rapidly changing availability of terminal electron acceptors without the need for transcriptional regulation and protein synthesis.

## Abstract (precis)

Thermophilic Gram-positive organisms were recently shown to be a promising class of organisms to be used in bioelectrochemical systems for the production of electrical energy. These organisms present a thick peptidoglycan layer that was thought to preclude them to perform extracellular electron transfer (i.e., exchange catabolic electrons with solid electron acceptors outside the cell). In this paper, we describe the structure and functional mechanisms of the multiheme cytochrome OcwA, the terminal reductase of the Gram-positive bacterium *Thermincola potens* JR found at the cell surface of this organism. The results presented here show that this protein can take the role of a respiratory “Swiss Army knife,” allowing this organism to grow in environments with soluble and insoluble substrates. Moreover, it is shown that it is unrelated to terminal reductases found at the cell surface of other electroactive organisms. Instead, OcwA is similar to terminal reductases of soluble electron acceptors. Our data reveal that terminal oxidoreductases of soluble and insoluble substrates are evolutionarily related, providing novel insights into the evolutionary pathway of multiheme cytochromes.

## INTRODUCTION

Iron is one of the most abundant metals in Earth’s crust, and the microbial reduction of iron is associated with some of the earliest life forms (1). Nowadays, this type of metabolism is used for numerous biotechnological processes, including the production of energy in microbial fuel cells (MFCs) and synthesis of added-value compounds in microbial electrosynthesis (MES) (2, 3). It is the ability to perform extracellular electron transfer that allows some microorganisms to exchange electrons with an electrode in these devices (4). While microbes in MFCs donate electrons to electrodes and generate electrical current (3), in MES, the electrode oxidation performed by bacteria is coupled to the production of chemicals on the cathode compartment (5). Currently, about 100 microorganisms are known to perform extracellular electron transfer and exchange electrons with an electrode (6). Most of these organisms are Gram-negative bacteria (6); this is mainly because of the long-held view that the thick peptidoglycan layer that encases Gram-positive bacteria prevents them from performing this type of metabolism (7). However, recently, it was described that Gram-positive bacteria are also able to perform extracellular electron transfer (8–10). The iron-reducing Gram-positive bacterium Thermincola potens JR was identified in current-producing MFCs operating at high temperature (11), whereas the closely related bacterium Thermincola ferriacetica was isolated from ferric deposits in a hydrothermal spring (12). These two thermophilic microorganisms (11) were shown to produce higher current levels in MFCs than with mesophilic organisms in the same type of bioreactor (11, 13). Despite their faster kinetics and lower interference from oxygen intrusion (13, 14), their application is still hindered by the lack of knowledge of the molecular mechanism they use for extracellular electron transfer. This has a negative impact on the ability to optimize bioelectrochemical systems for the efficient production of energy using this promising class of organisms.

Like Gram-negative electroactive organisms, Thermincola spp. contain a large number of genes that code for multiheme *c*-type cytochromes (MHCs) (11, 15). Recently, a putative electron transfer pathway was proposed for *T. potens* JR (9). In this pathway, the nonaheme cytochrome TherJR_2595 (Tfer_3193 in *T. ferriacetica*) was shown to be located at the cell surface and proposed to be the terminal reductase for extracellular electron transfer in this organism (9). The characterization of this protein is essential to elucidate the molecular mechanisms of electron transfer at the electrode-microbe interface in Gram-positive bacteria.

In this work, we describe the structural and functional properties of TherJR_2595, here referred to as OcwA (Outer cell wall protein A). The three-dimensional structure of this protein reveals that it is unrelated to the structurally characterized outer membrane MHCs from Shewanella spp. Instead, it is structurally and functionally related to MHCs involved in the biogeochemical cycles of nitrogen and sulfur, which suggests that terminal reductases that use soluble and insoluble electron acceptors may be evolutionarily related. This work provides the first insight into the molecular mechanisms of terminal reductases from thermophilic Gram-positive bacteria, which underpin the direct and indirect electron transfer to electrodes. This knowledge is crucial for the implementation of MFCs with a broader microbiological range and under more varied operational conditions, such as at high temperatures.

## RESULTS

### Production of OcwA.

**FIG 1:**
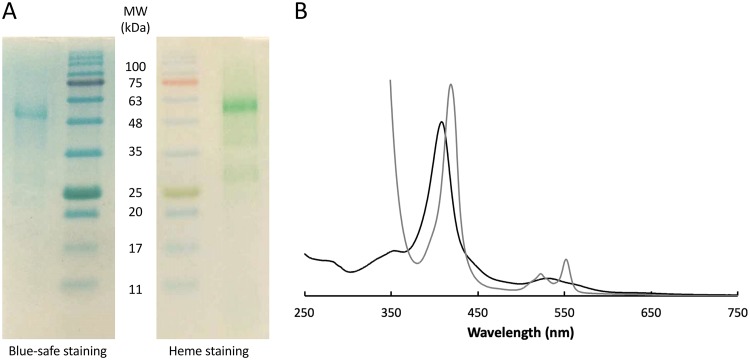
Isolation of OcwA from *T. potens* JR. (A) Blue-safe and heme-stained SDS-PAGE of purified OcwA. (B) UV-visible spectra of OcwA obtained in the reduced (gray) and oxidized (black) states.

**FIG 2:**
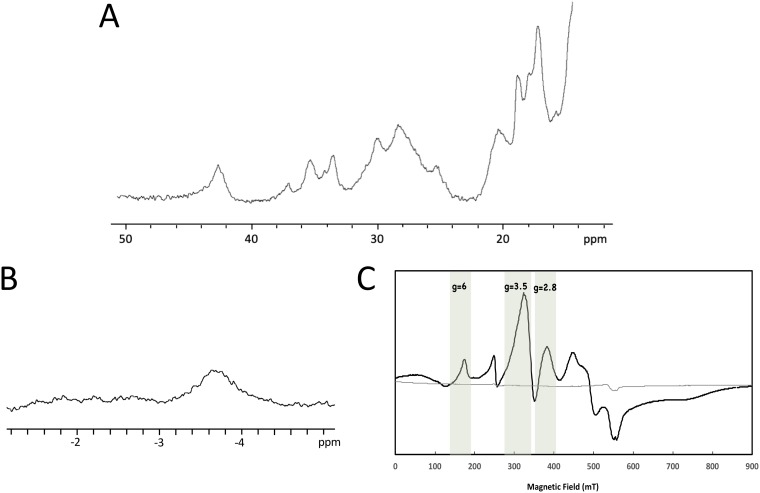
Magnetic spectroscopic properties of OcwA from *T. potens* JR. (A) ^1^H NMR spectrum of OcwA obtained at 25°C in the oxidized state (B) One-dimensional (1D) ^1^H NMR spectrum of OcwA in the reduced state obtained at 25°C by the addition of sodium dithionite. (C) EPR spectra of OcwA at 9.39 GHz in the oxidized (black line) and reduced (gray line) states at 7 K. The unlabeled signal at *g *of 4.3 likely corresponds to a small amount of high-spin ferric iron adventitiously present in the sample.

Recombinant OcwA was purified to electrophoretic homogeneity and identified as a band at approximately 62 kDa in SDS-PAGE (Fig. 1A). This band stained positively for covalently attached hemes (Fig. 1A), and N-terminal sequencing retrieved the predicted sequence of OcwA (EKPAD) without the signal peptide, showing that the protein was efficiently processed in Escherichia coli. Size exclusion chromatography of pure OcwA revealed an approximately equal mixture of monomer and dimer forms (see Fig. S1 in the supplemental material). UV-visible spectra of OcwA showed the typical features of a low-spin cytochrome *c* (Fig. 1B), and nuclear magnetic resonance (NMR) and electron paramagnetic resonance (EPR) spectroscopy indicated that this protein contains at least three types of hemes (Fig. 2).

10.1128/mBio.01210-19.2 FIG S1 Size exclusion chromatography of OcwA on a S200 10/300 GL (GE Healthcare) in 40 mm HEPES (pH 7.5) and 150 mM KCl. Download FIG S1, TIF file, 0.9 MB. Copyright © 2019 Costa et al. 2019 Costa et al. This content is distributed under the terms of the Creative Commons Attribution 4.0 International license.

The ^1^H NMR spectrum of OcwA in the oxidized state presents signals outside the protein envelope up to 45 ppm (Fig. 2A). These signals display a Curie-type temperature dependence (Fig. S2), which are typical of the methyl groups of low-spin ferric (Fe^3+^) hemes with two strong-field axial ligands, such as histidines or methionines (16). The reduction of OcwA with sodium dithionite revealed an NMR peak near −3 ppm (Fig. 2B). This indicates that besides the typical bishistidine axial coordinated hemes, OcwA also contains at least one heme that is axially coordinated by a methionine (17). Interestingly, this occurs even though the amino acid sequence of OcwA contains enough histidines for all nine hemes to be bishistidine coordinated.

10.1128/mBio.01210-19.3 FIG S2 Curie behavior of the ^1^H NMR chemical shifts shown in the spectrum. Download FIG S2, TIF file, 1.0 MB. Copyright © 2019 Costa et al. 2019 Costa et al. This content is distributed under the terms of the Creative Commons Attribution 4.0 International license.

Continuous-wave X-band EPR spectroscopy shows that reduced OcwA is silent, whereas the oxidized protein revealed a superposition of signals that suggest the presence of at least three groups of paramagnetic species (Fig. 2C). The shape of the spectrum shows clearly the presence of magnetic interactions among the hemes, as often observed in MHCs and expected due to the short distance between hemes in OcwA (as detailed in next section). The signal with a *g*_max_ of 2.8 is characteristic of low-spin (S = 1/2) six-coordinated hemes with axial ligands approximately parallel with each other, whereas signals with a *g*_max_ of 3.5 are due to low-spin six-coordinated hemes with axial ligands perpendicular to each other (18). The resonance at a *g* value of ∼6 indicates the presence of high-spin hemes (S = 5/2) that are typically five coordinated.

### Three-dimensional structure of OcwA.

**FIG 3:**
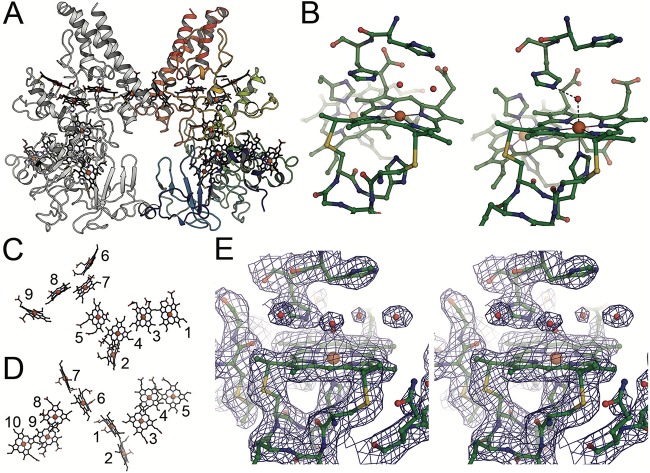
Structure of OcwA from *T*. potens JR. (A) Dimer structure of OcwA. The right monomer is colored from blue at the N terminus to red at the C terminus. Heme groups are depicted in a stick representation. (B) Close-up of the active-site heme groups 2 (left) and 5 (right) highlighting the similar environment of the two centers and depicting the water molecules found on the distal side. (C and D) Comparison of the heme cores of OcwA (C) and MtrC (D) (PDB 4LM8). (E) Stereo representation of the 2F_o_–F_c_ electron density map surrounding heme group 2, contoured at the 1 σ level.

The three-dimensional (3D) structure of OcwA was determined to 2.2-Å resolution by X-ray crystallography (Table S1). OcwA is composed by a globular heme domain and a three-helix bundle at the C terminus. These three long α-helices are reminiscent of the pentaheme NrfA (nitrite reductase) family of proteins (19) and of the octaheme cytochromes hydroxylamine oxidoreductase (HAO) and (20) sulfite reductase MccA (Fig. 3A) (21). The crystal structure of OcwA shows that this protein forms a dimer, as observed experimentally by gel filtration (Fig. S1). It spans about 180 by 81 by 45 Å but presents only limited contact (<2% of the total surface area) at the interface between monomers involving two regions. While the first contact area is formed by the base, but not the remainder, of the three-helix bundle, the second is made by a small alpha helix and a neighboring loop from an additional beta-sheet domain (amino acids 75 to 156) within the globular heme domain. This is an unprecedented monomer arrangement, where the three-helix bundle does not dominate the formation of the dimer interface (typical dimer interfaces are in the range of 8 to 12% of the surface of the monomer). This arrangement may be a consequence of the decreased length of the C-terminal helices with respect to NrfA proteins.

10.1128/mBio.01210-19.1 TABLE S1 Data collection and refinement statistics. Download Table S1, DOCX file, 0.1 MB. Copyright © 2019 Costa et al. 2019 Costa et al. This content is distributed under the terms of the Creative Commons Attribution 4.0 International license.

The hemes in OcwA present three distinct coordination environments, as follows: hemes 1, 3, 4, 6, 7, and 8 (numbered according to the position of the CXXCH binding motif in the polypeptide sequence, where X is any amino acid) are bishistidine coordinated. Among these, hemes 1, 3, and 6 likely contribute to the EPR signal, with a *g* value of 2.8, because their axial histidines have nearly parallel rings. In contrast, hemes 4, 7, and 8 likely contribute to the EPR signal, with a *g* value of 3.5, as their axial histidines have nearly perpendicular rings. Heme 9 features histidine-methionine coordination, whereas hemes 2 and 5 have histidine as a proximal ligand and an open coordination site at the distal position (Fig. 3B and E), thus being responsible for the EPR signal with a *g* value of 6. These two hemes, located at opposite ends of the nine-heme arrangement, have an almost identical environment, with a histidine serving as proximal iron ligand and a His-His motif (HH 281/282 at heme 2 and HH 380/381 at heme 5) situated above the distal face of the heme, which in both cases forms a solvent-exposed pocket. The presence of these two penta-coordinated hemes that work as two putative active sites for substrate binding within a single monomer is a novelty within the family of MHCs (22).

**FIG 4:**
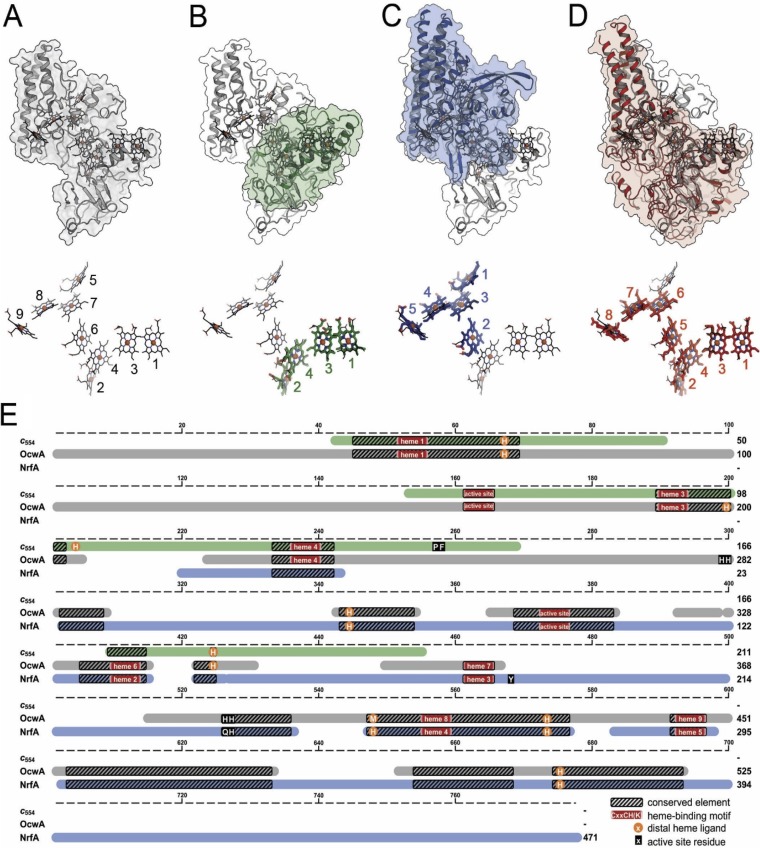
Structural and sequence similarities within the NrfA family of MHC. (A) Structure of the OcwA monomer and heme arrangement (below), with numbering of cofactors according to their occurrence in the protein sequence. (B) Structural alignment with Nitrosomonas europaea cytochrome *c*_554_ (PDB 1FT5) showing the heme numbering of this cytochrome. (C) Structural alignment with *Wollinella succinogenes* NrfA (PDB 1FS7) showing the heme numbering of this pentaheme cytochrome. (D) Structural alignment with *W. succinogenes* MccA (PDB 4RKM) showing the numbering of this octaheme cytochrome. (E) Schematic presentation of a structure-based amino acid sequence alignment of OcwA, NrfA, and c_554_, highlighting axial heme ligands (orange circles) and distal residues of the active site(s) (black boxes). Hatched boxes represent areas with high structural homology. Heme motifs are shown with red boxes.

The organization of the hemes within OcwA, although vaguely reminiscent of the “staggered-cross” design of the structurally characterized outer membrane MHCs (23, 24) (Fig. 3C and D), clearly follows a different design that is similar to that of the NrfA family of proteins, with alternating parallel and perpendicular diheme packing motifs (25). In fact, hemes 1 to 4 of OcwA align with a root mean square deviation (RMSD) for all atoms of 0.84 Å with the hemes of the tetraheme cytochrome *c*_554_ from Nitrosomonas europaea. In both proteins, heme 2 is the active site (Fig. 4B). Moreover, hemes 5 to 9 of OcwA can be superimposed, with an RMSD of 0.89 Å to the NrfA heme core structure (Fig. 4C), with heme 5 of OcwA as the second active site. Interestingly, hemes 1 to 4 and 6 to 9 also align with an RMSD of 0.89 Å to the heme core structure of the sulfite reductase MccA (21), with heme 2 in both proteins acting as the active site (Fig. 4D). Although there is some correspondence of the polypeptides of cytochrome *c*_554_, NrfA, and MccA with the corresponding sections of OcwA, sequence similarity is only observed for the heme-binding motifs, the distal heme ligands except for those of heme 2, and the three-helix bundle. Furthermore, MccA and OcwA share the additional β-sheet domain that has no equivalence in other NrfA family proteins (Fig. S3).

10.1128/mBio.01210-19.4 FIG S3 Alignment of OcwA with cytochrome *c*_554_, NrfA, and MccA highlighting areas of structural homology (black boxes), heme-binding motifs (red bars), axial heme ligands (orange arrows and boxes), and active-site residues (green arrow and boxes). Blue arrows on the sequence mark α-helices, and yellow arrows mark β-sheets. Download FIG S3, TIF file, 2.2 MB. Copyright © 2019 Costa et al. 2019 Costa et al. This content is distributed under the terms of the Creative Commons Attribution 4.0 International license.

### Electron transfer in OcwA.

**FIG 5:**
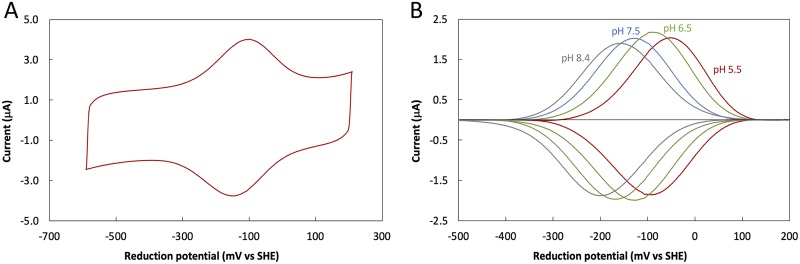
Cyclic voltammetry of OcwA. (A) Raw voltammogram obtained at a scan rate of 200 mV/s at pH 7.5. (B) Baseline-subtracted data of the voltammograms obtained at a scan rate of 200 mV/s at different pH values.

The redox behavior of OcwA was explored using cyclic voltammetry. The experimental configuration used closely mimics the physiological context of this protein that is located at the surface of *T. potens* JR and exchanges electrons with electrodes in MFCs. OcwA formed a stable film at the surface of the pyrolytic graphite “edge” electrode and displayed reversible electrochemistry over a wide potential window ranging from +100 mV (fully oxidized) to −450 mV (fully reduced) versus standard hydrogen electrode (SHE) (Fig. 5A). This potential window is similar to that previously observed for other terminal reductases present at the cell surface of other electroactive organisms (26). This range of potentials shows that OcwA may function as the terminal reductase of *T. potens* JR, being responsible for transferring electrons to the electrode. Indeed, the overall rate of interfacial electron transfer between OcwA and the electrode determined from trumpet plots (Fig. S4) is approximately 150 s^−1^, which is in the same range as those reported for other outer membrane terminal reductases (∼100 s^−1^) (27). Moreover, kinetic experiments with amorphous iron oxide, which is known to sustain the growth of *Thermincola* spp., showed that OcwA has the necessary reactivity to act as a terminal reductase in this organism (Fig. S5).

10.1128/mBio.01210-19.5 FIG S4 Positions of the oxidation and reduction peaks of the redox cofactors of OcwA of *T. potens* JR as a function of scan rate (log scale) for a range of different pHs (5.5 to 8.5). Fitting of the data (dashed lines) was obtained using the trumpet by function of QSoas (Fourmond [51]), which relies on numerical integration of the Laviron differential equations using the implicit Bulirsch-Stoer solver of Bader and Deuflhard (Bader and Deuflhard, 1983). Download FIG S4, TIF file, 1.1 MB. Copyright © 2019 Costa et al. 2019 Costa et al. This content is distributed under the terms of the Creative Commons Attribution 4.0 International license.

10.1128/mBio.01210-19.6 FIG S5 Reaction of OcwA with iron oxides. (A) Kinetic experiments measured at 418 nm. (B) UV-visible spectra of reduced OcwA before (black line) and after (blue line) the addition of iron (III) oxides. Download FIG S5, TIF file, 0.9 MB. Copyright © 2019 Costa et al. 2019 Costa et al. This content is distributed under the terms of the Creative Commons Attribution 4.0 International license.

Electrochemical titrations of OcwA followed by cyclic voltammetry showed the pH dependence of the signals (Fig. 5B), which is an indication that electron transfer is coupled to proton transfer in the physiological range (i.e., redox-Bohr effect) (28, 29). This allows redox proteins with multiple closely packed centers the possibility of becoming fully reduced by balancing the electrostatic repulsion that would arise from taking up multiple negative charges with the uptake of protons.

**FIG 6:**
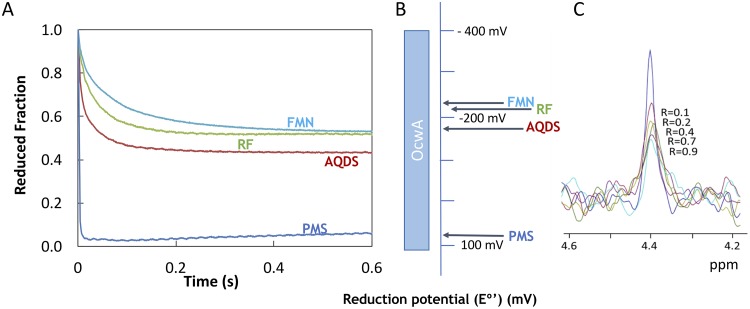
Reactivity of OcwA with electron shuttles. (A) Kinetic data obtained by mixing OcwA (0.43 μM) with excess of AQDS (36 μM), PMS (9 μM), FMN (19 μM), and RF (14 μM). (B) Midpoint reduction potentials of the different electron shuttles versus the redox-active range of OcwA at pH 7.6 (C) 1D ^31^P-NMR spectra of FMN in the presence of increasing amounts of OcwA obtained at 25°C.

Since *T. potens* JR was shown to use soluble electron shuttles (30), we tested the ability of OcwA to interact with anthraquinone-2,6-disulfonate (AQDS), flavin mononucleotide (FMN), riboflavin (RF), and phenazine methosulfate (PMS). These electron shuttles are typically found in environments associated with electroactive microorganisms, and AQDS is known to support the growth of *T. potens* JR (11). Stopped-flow experiments showed that OcwA is oxidized by the four electron shuttles tested (Fig. 6A). The degree of oxidation of OcwA at the end of the experiment is indicative of a thermodynamic equilibrium between the protein and the electron shuttles, where the different endpoints observed correlate with the midpoint reduction potential of the various electron shuttles (Fig. 6B).

To assess the binding of the electron shuttles to OcwA, we used ^1^H NMR spectroscopy. The comparison of the high-frequency region spectra obtained for OcwA alone and after the addition of increasing amounts of the electron shuttles revealed no perturbation of the methyl signals located outside the protein envelope arising from low-spin hemes (Fig. S6). This indicates that the binding of the electron shuttles may occur away from these methyl groups. This is, however, in contrast with the observation for the outer membrane MHCs from *Shewanella* spp., where the binding of soluble redox shuttles to MtrC, OmcA, and UndA caused a disturbance of the heme methyl signals (31).

10.1128/mBio.01210-19.7 FIG S6 ^1^H NMR spectrum of OcwA obtained at 25°C in the oxidized state before (red) and after (black) the addition of AQDS. Download FIG S6, TIF file, 1.6 MB. Copyright © 2019 Costa et al. 2019 Costa et al. This content is distributed under the terms of the Creative Commons Attribution 4.0 International license.

Taking advantage of the phosphorus atom contained in FMN, ^31^P NMR experiments were used to monitor the binding of this redox shuttle to OcwA. With increasing amounts of OcwA, we observed that the FMN phosphorus signal broadens and diminishes in intensity (Fig. 6C). These spectral changes arise from binding of FMN to OcwA in a slow exchange regime on the NMR time scale, which suggests strong binding. This behavior is also different from what was previously reported for the outer membrane MHCs from Shewanella oneidensis MR-1, where the binding was transient and occurred in the fast regime on the NMR time scale (31).

### Catalytic activity of OcwA.

Prompted by the structural homology with the NrfA family of proteins, reactivities with nitrite, hydroxylamine, and sulfite were tested, given that these are the most prevalent substrates of this type of enzymes (Fig. S7). It was observed that OcwA can reduce nitrite, but not sulfite, under the conditions tested. The reduction and oxidation of hydroxylamine were also observed.

10.1128/mBio.01210-19.8 FIG S7 (A to D) Reaction of OcwA with different soluble substrates by monitoring methyl viologen oxidation at 732 nm over time (A to C) and MTT reduction at 578 nm (D). Blue line is upon the addition of OcwA, while black line is the control assay, where buffer, instead of OcwA, was added. (A) Reduction of nitrite. (B) Reduction of sulfite. (C and D) Reduction of hydroxylamine (C) and oxidation of hydroxylamine (D). Download FIG S7, TIF file, 1.5 MB. Copyright © 2019 Costa et al. 2019 Costa et al. This content is distributed under the terms of the Creative Commons Attribution 4.0 International license.

For nitrite, *k*_cat_ values of 1 min^−1^ and *K_M_* of 65 μM were obtained, which are significantly lower than those reported for the bona fide nitrite reductase pentaheme NrfA (25). The lack of reduction of sulfite may be explained by the lack of a copper atom, which although not crucial for sulfite reduction activity (32), is present in the active site of the homologous MccA (21).

## DISCUSSION

The importance of MHCs in extracellular electron transfer pathways of electroactive organisms has made their structural and functional characterization a priority in the bioelectromicrobiology field. This knowledge is even more significant for terminal reductases that are present at the cell surface of these organisms, where the knowledge on their mode of action is important to optimize the electron transfer process at the microbe-electrode interface (33, 34).

Surface-exposed OcwA is proposed to be the terminal reductase in *T. potens* JR extracellular electron transfer pathway, where it bridges the contact between bacterial metabolism and electrodes in MFC (9). BLASTP sequencing revealed an OcwA homolog in *T. ferriacetica* (Tfer_3193), with which it shares 99% identity (520/525 amino acids), including heme-binding motifs and axial ligands (Fig. S8), thus suggesting a similar role between both proteins from different species of *Thermincola*. Given that a genetic system has not been developed for *Thermincola* spp., the generation of knockout strains that would confirm this hypothesis is still not possible. Nonetheless, here, we have shown that OcwA from *T. potens* JR fulfills the role of a terminal reductase for extracellular electron transfer. It is capable of exchanging electrons with electrodes at rates similar to those of the surface-exposed cytochromes of *Shewanella* spp., which were previously characterized, and to reduce iron oxides.

10.1128/mBio.01210-19.9 FIG S8 Alignment of the amino acid sequence of OcwA from *T. potens* JR (TherJR_2595) and from *T. ferriacetica* (Tferr_3193). The boxes show the heme-binding site of both proteins. Download FIG S8, TIF file, 2.1 MB. Copyright © 2019 Costa et al. 2019 Costa et al. This content is distributed under the terms of the Creative Commons Attribution 4.0 International license.

The redox-Bohr effect presented by OcwA appears to match the needs of *Thermincola* spp. when growing as a biofilm at the surface of an electrode. As demonstrated by Lusk and coworkers upon studying the rate-limiting enzymatic response responsible for the electrochemical signal of *T. ferriacetica* (35), one of the key factors for the stability of electrochemically active biofilms at the surface of electrodes is the efficient dissipation of pH gradients to maintain the viability of the cells (36) and, therefore, the current generation (13). A similar proton-electron coupling is also found in the cell surface-exposed MHCs from *Shewanella* spp. (26, 37). Also, similar to the MHCs from *Shewanella* spp., OcwA shows an internal organization of the hemes that is neither globular nor linear but instead branched (24). This suggests that for the MHCs found at the surface of electroactive bacteria, having multiple hemes that can work as entry and exit electron points is an important design feature.

However, it is at the level of the differences with previously characterized MHCs found at the surface of *Shewanella* spp. that OcwA expands our current understanding of nature to efficiently perform extracellular electron transfer. OcwA shows for the first time in cell surface MHCs a variety of heme coordination environments, including the open distal axial coordination positions of hemes 2 and 5. High-spin hemes are typically associated with active sites where substrates are bound and chemical reactions take place. Interestingly, the heme-binding motif of the high-spin hemes 2 and 5 in OcwA are not identical to the catalytic heme of most known NrfA proteins (i.e., CXXCK motif), nor to the P460 catalytic heme of hydroxylamine oxidizing HAO, even though OcwA can perform both reactions. Although organisms of *Thermincola* were never reported to grow in the presence of nitrite or hydroxylamine (11), the ability of OcwA to reduce these substrates may be advantageous for cell survival in anaerobic environments. Indeed, OcwA may work as a detoxifying enzyme when these organisms encounter these compounds, as observed for other MHCs (38), which is a novelty for cell surface terminal reductases. Furthermore, OcwA interacts with soluble electron shuttles, namely, FMN, in a way that is distinct from its counterparts from S. oneidensis, OmcA and MtrC, whose interaction has been proposed to be transient and modulated by redox-active disulfide bridges (35, 39). In fact, OcwA not only lacks such bridges but also binds strongly to FMN.

Finally, rather than being a truncated member of the MtrC/OmcA/UndA protein family lacking one heme, OcwA is structurally related to NrfA, cytochrome *c*_554_, and MccA, which are key proteins in the nitrogen and sulfur cycles. It was proposed that the octaheme cytochrome *c* family evolved from the duplication of an ancestral *nrfA* gene in deltaproteobacteria, which was then extended by fusion with a triheme cytochrome *c* (22). The structure and catalytic versatility of OcwA provide a more nuanced view of this evolutionary storyline, now in the context of Gram-positive bacteria. From the structural point of view, OcwA can be described as a pentaheme cytochrome fused to the C terminus of the tetrahemic scaffold of cytochrome *c*_554_. Indeed, cytochromes of the NrfA, *c*_554_, and octaheme families are evolutionarily related (2, 22). The structure of OcwA raises the possibility that the extant octaheme cytochromes evolved by the loss of heme 2 (HAO) or heme 5 (MccA) from ancestral nonaheme cytochromes similar to OcwA.

In conclusion, the structural and functional characterization of OcwA reveals that this terminal reductase for extracellular electron transfer is unrelated to the known terminal reductases MtrC, OmcA, and UndA. This indicates that the process of extracellular electron transfer evolved independently more than once to accommodate the catabolic needs of microorganisms living in environments with access to solid terminal electron acceptors. Moreover, the particular case of OcwA reveals a multifunctional link between the biogeochemical cycles of nitrogen and iron. Indeed, being able to react with electrodes, N-oxides, iron, and small-molecule redox shuttles, OcwA may work as the respiratory “Swiss Army knife” of *Thermincola* spp., allowing this Gram-positive bacterium to grow and survive in environments with varied access to solid and soluble electron acceptors.

## MATERIALS AND METHODS

### Protein production and purification.

The OcwA protein was produced according to the literature (40), with minor changes, using the primers listed in Table 1. Briefly, a chimeric gene containing the signal peptide of small tetraheme cytochrome c (*stc*) from S. oneidensis MR-1 fused with the gene sequence of *therjr_2595* without native signal peptide was constructed. E. coli JM109(DE3) was cotransformed with this chimeric gene previously cloned into the pBAD202/d-TOPO vector (41) and plasmid pEC86 that contains the cytochrome *c* maturation system (42). Transformed E. coli strains were cultured in TB medium with 50 μg kanamycin and 35 μg of chloramphenicol at 37°C for 6 h. At mid-log phase (∼6 h after inoculation), the temperature of the growing culture in TB medium previously set at 37°C was lowered to 30°C. The cells were allowed to grow for additional 18 h, pelleted by centrifugation at 10,000 × *g* for 10 min at 4°C, and resuspended in osmotic shock solution (0.2 M Tris-HCl [pH 7.6] with 0.5 M sucrose, 0.5 mM EDTA, and 100 mg/liter lysozyme) with protease inhibitor (Sigma) and DNase (Sigma) using a ratio of 200 ml of osmotic shock solution for each 8 liters of culture medium. The spheroplast solution was incubated at 4°C for 40 min with gentle stirring. The periplasmic fraction containing the recombinant protein was cleared by ultracentrifugation at 200,000 × *g* for 1 h at 4°C and dialyzed overnight against 20 mM Tris-HCl (pH 7.6). The dialyzed protein extract was purified via a HisTrap column (GE Healthcare) previously equilibrated with 20 mM sodium phosphate buffer, 20 mM imidazole, and 500 mM NaCl (pH 7.6). To elute the bound protein, a single step was used with 20 mM sodium phosphate buffer, 300 mM imidazole, and 500 mM NaCl (pH 7.6). The resulting fraction was dialyzed overnight against 20 mM Tris-HCl (pH 7.6). A DEAE-biogel column (Bio-Rad) preequilibrated with 20 mM Tris-HCl (pH 7.6) was used as a final purification step. OcwA was eluted in 20 mM Tris-HCl buffer with 120 mM NaCl (pH 7.6). The purified protein was dialyzed against 20 mM potassium phosphate buffer (pH 7.6) with 100 mM KCl. SDS-PAGE (12% resolving gel) and UV-visible spectroscopy were used after each purification step to select the fractions containing the target protein and to evaluate its purity. The purified protein had an absorbance ratio (*A*_Soret peak_/*A*_280_) larger than 5.0.

**TABLE 1:** Oligonucleotides used to construct the chimeric gene

Primer	Sequence
STC_pBAD Forw	CACCTAAGAAGGAGATATACATCCCGTGAGCAAAAAACTATTAAG
pBAD_2595 Rev	TTGGAGTTTCTGTTCCGCAGCGGTTAACAGCTTAACG
STC_2595 Forw	CCAACCGCATTTGCCGAAAAGCCTGCGGACA
STC_2595 Rev	TGTCCGCAGGCTTTTCGGCAAATGCGGTTGG

### Crystallization and structure determination.

For structure determination, crystals were grown in an anoxic chamber (Coy) under an N_2_-H_2_ atmosphere (95%/5% [vol/vol]) at room temperature. Sitting drop vapor diffusion experiments were set up by adding 0.5 μl of protein solution (6 mg/ml) to 0.5 μl of reservoir solution. Well-diffracting crystals were obtained under conditions containing 24 to 27% polyethylene glycol 3350, 0.1 M HEPES (pH 7 to 7.5), and 0.1 to 0.2 M MgCl_2_ only after seeding. The crystals were harvested using 10% 2,3-butanediol as a cryoprotectant in mother liquor and flash frozen in liquid nitrogen. Data sets were collected at the Swiss Light Source, Paul-Scherrer-Institut (Villigen, Switzerland) on beamline X06DA with a Pilatus 2M detector (Dectris). Initial phase information was obtained by single anomalous diffraction with a data set collected at the iron edge, λ = 1.729 Å, using the PHENIX suite (43) for automatic phasing and initial model building. An initial structural model was refined in iterative steps using REFMAC5 (44) and Coot (45) to 2.7-Å resolution. This solution was then used for molecular replacement with *MOLREP* (46) as part of the CCP4 suite (47) for the more highly resolved structure at 2.2 Å. The final model comprised residues 42 to the very C terminus at residue 525 in chain A and residues 43 to 525 in chain B. Coordinates and structure factors have been deposited with the Protein Data Bank under the accession code 6I5B. Data collection and refinement statistics are summarized in Table S1.

### NMR experiments.

For all of the NMR experiments, OcwA prepared in 20 mM potassium phosphate buffer (pH 7.6) with 100 mM KCl was lyophilized and resuspended in D_2_O. An excess of sodium dithionite was used to reduce the protein. ^1^H NMR experiments were performed on a Bruker Avance II 500 MHz spectrometer equipped with a QXI probe for ^1^H detection and an SEX probe for ^31^P detection. All NMR data were processed in the TopSpin 3.2 software. ^1^H NMR spectra were acquired before and after lyophilization to ensure that protein integrity was preserved.

To study the influence of the electron shuttles on OcwA, NMR experiments were performed as previously described (31) using antraquinone-2,6-disulfonate (AQDS), flavin mononucleotide (FMN), riboflavin (RF), and phenazine methosulfate (PMS). Stock solutions of the different electron shuttles were prepared in 20 mM potassium phosphate buffer (pH 7.6) with 100 mM KCl. ^1^H NMR spectra performed at 25°C on a Bruker Avance II 500 MHz NMR spectrometer equipped with a TCI cryoprobe for ^1^H detection were acquired before and after the addition of the electron shuttles (molar ratios 0.5:1, 1:1, and 3:1 of electron shuttle to protein).

For the ^31^P-NMR binding experiments, samples containing 100 μM FMN prepared in 20 mM phosphate buffer (pH 7.6) with 100 mM KCl were titrated against increasing concentrations of OcwA at 25°C.

### EPR experiments.

The OcwA solution was prepared in 20 mM potassium phosphate buffer (pH 7.6) with 100 mM KCl to a final concentration of 200 μM. The reduced state was obtained by the addition of an excess of sodium borohydride. EPR spectra were recorded on a Bruker ESP 380 spectrometer equipped with an ESR 900 continuous-flow helium cryostat (Oxford Instruments). The conditions were temperature, 7 K; microwave frequency, 9.39 GHz; modulation amplitude, 1.0 mT; and microwave power, 2 mW.

### Cyclic voltammetry of OcwA.

The electrochemical setup was assembled as previously described (48, 49). Electrochemical measurements were performed in an anaerobic glove box (Jacomex, France) with a nitrogen atmosphere (residual O_2_, <1 ppm). A pyrolytic graphite edge (PGE) electrode (surface, approximately 3 mm^2^) was polished with alumina slurry (1 μm; Buehler) and then coated with 0.5 μl of a solution of OcwA (stock solution previously diluted in 20 mM potassium phosphate buffer [pH 7.6] with 100 mM KCl to a final concentration of 50 μM) and left to dry for approximately 5 min. The buffers used in the electrochemical experiments were prepared by mixing 5 mM HEPES, MES, *N*-Tris(hydroxymethyl)methyl-3-aminopropanesulfonic acid (TAPS), and 100 mM KCl. The desired pH values were adjusted with 1 M NaOH or HCl solution. Experiments were performed at 25°C using an electrochemical cell consisting of an Ag/AgCl (saturated KCl) reference electrode in a Luggin sidearm and a platinum wire counter electrode. Cyclic voltammetry was performed with an Autolab electrochemical analyzer (PGSTAT-128N) with an analogue scan generator controlled by the GPES software. The potentials are quoted with reference to standard hydrogen electrode (SHE) by the addition of 0.197 V to those measured (50). The electrochemical data were analyzed using the QSoas program available at http://bip.cnrs-mrs.fr/bip06/qsoas/ (51).

### Kinetic experiments between OcwA and electron shuttles.

Protein oxidation experiments were performed according to Paquete et al. (31) in a stopped-flow apparatus (SHU-61VX2; TgK Scientific) placed inside an anaerobic chamber (M-Braun 150) containing less than 5 ppm of oxygen. The concentration of protein, approximately 0.4 μM after mixing, was determined for each experiment by UV-visible spectroscopy using ε_409nm_ of 125.000 M^−1^ cm^−1^ per heme for the oxidized state of the protein (49, 52). Stock solutions (5 mM) of the electron shuttles antraquinone-2,6-disulfonate (AQDS), flavin mononucleotide (FMN), riboflavin (RF), and phenazine methosulfate (PMS) were prepared by dissolving weighted amounts of solid reagents in 20 mM potassium phosphate buffer (pH 7.6) with 100 mM KCl. Dilutions of the electron shuttles were prepared in degassed buffer, and their concentrations were determined by UV-visible spectroscopy using the following extinction coefficients: ε_326nm_ of 5,200 M^−1^ cm^−1^ for AQDS (53), ε_445nm_ of 12,200 M^−1^ cm^−1^ for FMN (54), ε_445nm_ of 12,500 M^−1^ cm^−1^ for RF (55), and ε_387nm_ of 26,300 M^−1^ cm^−1^ for PMS (56). Reduced OcwA was obtained by mixing the protein with small volumes of concentrated sodium dithionite solution. UV-visible spectroscopy was used to confirm that there was no excess of dithionite using ε_314nm_ of 8,000 M^−1^ cm^−1^ (57). Oxidation by electron shuttles was monitored by measuring the absorption changes at 552 nm upon mixing reduced OcwA with each of them. The temperature of the kinetic experiments was maintained at 25°C using an external circulating bath (31).

### Catalytic experiments with OcwA.

The enzymatic reduction of sulfite, nitrite, and hydroxylamine by OcwA was tested at 55°C in an anaerobic chamber (Coy). All of the assays were performed in 50 mM potassium phosphate (KPi) buffer at pH 7.0, with up to 1 mM methyl viologen previously reduced with zinc granules, as described in the literature (32). The reaction was started by the addition of the substrate, followed by OcwA, and the reaction was monitored by the oxidation of methyl viologen at 732 nm using a Shimadzu UV-1800 spectrophotometer. Sodium sulfite concentrations were tested in the range of 100 μM to 6 mM, while sodium nitrite was tested in the range 20 μM to 150 μM and hydroxylamine in the range 10 μM to 200 μM. The final concentrations of OcwA ranged from 50 to 400 nM measured prior to the experiments using ε_409_ nm of 125.000 M^−1^ cm^−1^ per heme for the oxidized state of the protein.

The enzymatic oxidation of hydroxylamine by OcwA was tested using 20 μM PMS and 400 μM 3-(4,5-dimethyl-2-thiazolyl)-2,5-diphenyl-2H-tetrazolium bromide (MTT) as electron acceptors (20). The reaction was started by the addition of hydroxylamine (75 μM to 500 μM), followed by 100 μM OcwA, and the reduction of MTT was monitored at 578 nm.

The enzymatic reduction of amorphous iron (III) oxide by OcwA was also performed at 55°C in the same anaerobic chamber. Amorphous iron (III) oxide was prepared as described by Zavarzina et al. (12), a solution of FeCl_3_ was titrated with 10% (wt/vol) NaOH (pH 11), and brown precipitate was formed. In each assay, iron (III) oxide was added to 488 nM OcwA, previously reduced with sodium dithionite, in 50 mM potassium phosphate buffer (pH 7.0). Iron (III) oxides were tested in the range of 14.3 to 55.5 mg/liter. UV-visible spectra of OcwA were obtained as isolated (oxidized state), after the addition of sodium dithionite (reduced state), and after addition of the iron oxides. All solutions were previously degassed.

## References

[1] Melton ED, Swanner ED, Behrens S, Schmidt C, Kappler A 2014 The interplay of microbially mediated and abiotic reactions in the biogeochemical Fe cycle. Nat Rev Microbiol 12:797–808. doi:10.1038/nrmicro3347. 25329406

[2] Iverson TM, Arciero DM, Hooper AB, Rees DC 2001 High-resolution structures of the oxidized and reduced states of cytochrome c_554_ from *Nitrosomonas europaea*. J Biol Inorg Chem 6:390–397. doi:10.1007/s007750100213. 11372197

[3] Iverson TM, Arciero DM, Hsu BT, Logan MSP, Hooper AB, Rees DC 1998 Heme packing motifs revealed by the crystal structure of the tetra-heme cytochrome c_554_ from Nitrosomonas europaea. Nat Struct Mol Biol 5:1005–1012. doi:10.1038/2975. 9808046

[4] Costa NL, Clarke TA, Philipp LA, Gescher J, Louro RO, Paquete CM 2018 Electron transfer process in microbial electrochemical technologies: the role of cell-surface exposed conductive proteins. Bioresour Technol 255:308–317. doi:10.1016/j.biortech.2018.01.133. 29444758

[5] Karthikeyan R, Singh R, Bose A 2019 Microbial electron uptake in microbial electrosynthesis : a mini‑review. J Ind Microbiol Biotechnol, in press. 10.1007/s10295-019-02166-6 30923971

[6] Koch C, Harnisch F 2016 Is there a specific ecological niche for electroactive microorganisms? ChemElectroChem 3:1282–1295. doi:10.1002/celc.201600079.

[7] Ehrlich HL 2008 Are gram-positive bacteria capable of electron transfer across their cell wall without an externally available electron shuttle? Geobiology 6:220–224. doi:10.1111/j.1472-4669.2007.00135.x. 18498525

[8] Light SH, Su L, Rivera-Lugo R, Cornejo JA, Louie A, Iavarone AT, Ajo-Franklin CM, Portnoy DA 2018 A flavin-based extracellular electron transfer mechanism in diverse Gram-positive bacteria. Nature 562:140–144. doi:10.1038/s41586-018-0498-z. 30209391 PMC6221200

[9] Carlson HK, Iavarone AT, Gorur A, Yeo BS, Tran R, Melnyk RA, Mathies RA, Auer M, Coates JD 2012 Surface multiheme *c*-type cytochromes from *Thermincola potens* and implications for respiratory metal reduction by Gram-positive bacteria. Proc Natl Acad Sci U S A 109:1702–1707. doi:10.1073/pnas.1112905109. 22307634 PMC3277152

[10] Gavrilov SN, Lloyd JR, Kostrikina NA, Slobodkin AI 2012 Fe(III) oxide reduction by a Gram-positive thermophile: physiological mechanisms for dissimilatory reduction of poorly crystalline Fe(III) Oxide by a thermophilic Gram-positive bacterium *Carboxydothermus ferrireducens*. Geomicrobiol J 29:804–819. doi:10.1080/01490451.2011.635755.

[11] Wrighton KC, Agbo P, Warnecke F, Weber KA, Brodie EL, DeSantis TZ, Hugenholtz P, Andersen GL, Coates JD 2008 A novel ecological role of the Firmicutes identified in thermophilic microbial fuel cells. ISME J 2:1146–1156. doi:10.1038/ismej.2008.48. 18769460

[12] Zavarzina DG, Sokolova TG, Tourova TP, Chernyh NA, Kostrikina NA, Bonch-Osmolovskaya EA 2007 Thermincola ferriacetica sp. nov., a new anaerobic, thermophilic, facultatively chemolithoautotrophic bacterium capable of dissimilatory Fe(III) reduction. Extremophiles 11:1–7. doi:10.1007/s00792-006-0004-7. 16988758

[13] Parameswaran P, Bry T, Popat SC, Lusk BG, Rittmann BE, Torres CI 2013 Kinetic, electrochemical, and microscopic characterization of the thermophilic, anode-respiring bacterium Thermincola ferriacetica. Environ Sci Technol 47:4934–4940. doi:10.1021/es400321c. 23544360

[14] Lusk BG 2019 Thermophiles; or, the modern Prometheus: the importance of extreme microorganisms for understanding and applying extracellular electron transfer. Front Microbiol 10:818. doi:10.3389/fmicb.2019.00818. 31080440 PMC6497744

[15] White GF, Edwards MJ, Gomez-Perez L, Richardson DJ, Butt JN, Clarke TA 2016 Mechanisms of bacterial extracellular electron exchange, p 87–138. *In* Poole R (ed), Advances in microbial physiology. Elsevier, London, United Kingdom. 10.1016/bs.ampbs.2016.02.002 27134022

[16] Louro RO, Correia IJ, Brennan L, Coutinho IB, Xavier AV, Turner DL 1998 Electronic structure of low-spin ferric porphyrins: ^13^C NMR studies of the influence of axial ligand orientation. J Am Chem Soc 120:13240–13247. doi:10.1021/ja983102m.

[17] Xavier AV, Czerwinski EW, Bethge PH, Mathews FS 1978 Identification of the haem ligands of cytochrome *b*_562_ by x-ray and NMR methods. Nature 275:245. doi:10.1038/275245a0. 357988

[18] Palmer G 1985 The electron paramagnetic resonance of metalloproteins. Biochem Soc Trans 13:548–560. doi:10.1042/bst0130548. 2993061

[19] Einsle O, Stach P, Messerschmidt A, Simon J, Kröger A, Huber R, Kroneck PM 2000 Cytochrome *c* nitrite reductase from *Wolinella succinogenes*. Structure at 1.6 A resolution, inhibitor binding, and heme-packing motifs. J Biol Chem 275:39608–39616. doi:10.1074/jbc.M006188200. 10984487

[20] Haase D, Hermann B, Einsle O, Simon J 2017 Epsilonproteobacterial hydroxylamine oxidoreductase (εHao): characterization of a ‘missing link’ in the multihaem cytochrome c family. Mol Microbiol 105:127–138. doi:10.1111/mmi.13690. 28388834

[21] Hermann B, Kern M, La Pietra L, Simon J, Einsle O 2015 The octahaem MccA is a haem *c*-copper sulfite reductase. Nature 520:706–709. doi:10.1038/nature14109. 25642962

[22] Klotz MG, Schmid MC, Strous M, Op Den Camp HJM, Jetten MSM, Hooper AB 2008 Evolution of an octahaem cytochrome c protein family that is key to aerobic and anaerobic ammonia oxidation by bacteria. Environ Microbiol 10:3150–3163. doi:10.1111/j.1462-2920.2008.01733.x. 18761666

[23] Edwards MJ, Fredrickson JK, Zachara JM, Richardson DJ, Clarke TA 2012 Analysis of structural MtrC models based on homology with the crystal structure of MtrF. Biochem Soc Trans 40:1181–1185. doi:10.1042/BST20120132. 23176451

[24] Clarke TA, Edwards MJ, Gates AJ, Hall A, White GF, Bradley J, Reardon CL, Shi L, Beliaev AS, Marshall MJ, Wang Z, Watmough NJ, Fredrickson JK, Zachara JM, Butt JN, Richardson DJ 2011 Structure of a bacterial cell surface decaheme electron conduit. Proc Natl Acad Sci U S A 108:9384–9389. doi:10.1073/pnas.1017200108. 21606337 PMC3111324

[25] Einsle O 2011 Structure and function of formate-dependent cytochrome c nitrite reductase, NrfA. Methods Enzymol 496:399–422. doi:10.1016/B978-0-12-386489-5.00016-6. 21514473

[26] Firer-Sherwood M, Su Pulcu G, Elliott SJ 2008 Electrochemical interrogations of the Mtr cytochromes from *Shewanella*: opening a potential window. J Biol Inorg Chem 13:849–854. doi:10.1007/s00775-008-0398-z. 18575901

[27] Baron D, LaBelle E, Coursolle D, Gralnick JA, Bond DR 2009 Electrochemical measurement of electron transfer kinetics by *Shewanella oneidensis MR-1*. J Biol Chem 284:28865–28873. doi:10.1074/jbc.M109.043455. 19661057 PMC2781432

[28] Louro RO, Catarino T, Salgueiro CA, LeGall J, Xavier AV 1996 Redox-Bohr effect in the tetrahaem cytochrome c_3_ from *Desulfovibrio vulgaris*: A model for energy transduction mechanisms. J Biol Inorg Chem 1:34–38. doi:10.1007/s007750050020.

[29] Papa S, Guerrieri F, Izzo G 1979 Redox Bohr-effects in the cytochrome system of mitochondria. FEBS Lett 105:213–216. doi:10.1016/0014-5793(79)80614-6. 39781

[30] Wrighton KC, Thrash JC, Melnyk RA, Bigi JP, Byrne-Bailey KG, Remis JP, Schichnes D, Auer M, Chang CJ, Coates JD 2011 Evidence for direct electron transfer by a Gram-positive bacterium isolated from a microbial fuel cell. Appl Environ Microbiol 77:7633–7639. doi:10.1128/AEM.05365-11. 21908627 PMC3209153

[31] Paquete CM, Fonseca BM, Cruz DR, Pereira TM, Pacheco I, Soares CM, Louro RO 2014 Exploring the molecular mechanisms of electron shuttling across the microbe/metal space. Front Microbiol 5:318. doi:10.3389/fmicb.2014.00318. 25018753 PMC4073285

[32] Lukat P, Rudolf M, Stach P, Messerschmidt A, Kroneck PMH, Simon J, Einsle O 2008 Binding and reduction of sulfite by cytochrome c nitrite reductase. Biochemistry 47:2080–2086. doi:10.1021/bi7021415. 18201106

[33] Rosenbaum MA, Henrich AW 2014 Engineering microbial electrocatalysis for chemical and fuel production. Curr Opin Biotechnol 29:93–98. doi:10.1016/j.copbio.2014.03.003. 24709348

[34] TerAvest MA, Ajo-Franklin CM 2016 Transforming exoelectrogens for biotechnology using synthetic biology. Biotechnol Bioeng 113:687–697. doi:10.1002/bit.25723. 26284614

[35] Lusk BG, Peraza I, Albal G, Marcus AK, Popat SC, Torres CI 2018 pH dependency in anode biofilms of Thermincola ferriacetica suggests a proton-dependent electrochemical response. J Am Chem Soc 140:5527–5534. doi:10.1021/jacs.8b01734. 29649873

[36] Ki D, Popat SC, Rittmann BE, Torres CI 2017 H_2_O_2_ production in microbial electrochemical cells fed with primary sludge. Environ Sci Technol 51:6139–6145. doi:10.1021/acs.est.7b00174. 28485588

[37] Hartshorne RS, Jepson BN, Clarke TA, Field SJ, Fredrickson J, Zachara J, Shi L, Butt JN, Richardson DJ 2007 Characterization of Shewanella oneidensis MtrC: a cell-surface decaheme cytochrome involved in respiratory electron transport to extracellular electron acceptors. J Biol Inorg Chem 12:1083–1094. doi:10.1007/s00775-007-0278-y. 17701062

[38] Simon J 2002 Enzymology and bioenergetics of respiratory nitrite ammonification. FEMS Microbiol Rev 26:285–309. doi:10.1111/j.1574-6976.2002.tb00616.x. 12165429

[39] Edwards MJ, White GF, Norman M, Tome-Fernandez A, Ainsworth E, Shi L, Fredrickson JK, Zachara JM, Butt JN, Richardson DJ, Clarke TA 2015 Redox linked flavin sites in extracellular decaheme proteins involved in microbe-mineral electron transfer. Sci Rep 5:11677. doi:10.1038/srep11677. 26126857 PMC4486940

[40] Costa NL, Carlson HK, Coates JD, Louro RO, Paquete CM 2015 Heterologous expression and purification of a multiheme cytochrome from a Gram-positive bacterium capable of performing extracellular respiration. Protein Expr Purif 111:48–52. doi:10.1016/j.pep.2015.03.007. 25797208

[41] Shi L, Lin J, Markillie LM, Squier TC, Brian S 2005 Overexpression of multi-heme *c*-type cytochromes. Biotechniques 38:297–299. doi:10.2144/05382PT01. 15727136

[42] Arslan E, Schulz H, Zufferey R, Künzler P, Thöny-Meyer L 1998 Overproduction of the Bradyrhizobium japonicum c-type cytochrome subunits of the cbb_3_ oxidase in *Escherichia coli*. Biochem Biophys Res Commun 251:744–747. doi:10.1006/bbrc.1998.9549. 9790980

[43] Afonine PV, Grosse-Kunstleve RW, Echols N, Headd JJ, Moriarty NW, Mustyakimov M, Terwilliger TC, Urzhumtsev A, Zwart PH, Adams PD 2012 Towards automated crystallographic structure refinement with phenix.refine. Acta Crystallogr D Biol Crystallogr 68:352–367. doi:10.1107/S0907444912001308. 22505256 PMC3322595

[44] Murshudov GN, Skubák P, Lebedev AA, Pannu NS, Steiner RA, Nicholls RA, Winn MD, Long F, Vagin AA 2011 REFMAC5 for the refinement of macromolecular crystal structures. Acta Crystallogr D Biol Crystallogr 67:355–367. doi:10.1107/S0907444911001314. 21460454 PMC3069751

[45] Emsley P, Lohkamp B, Scott WG, Cowtan K 2010 Features and development of Coot. Acta Crystallogr D Biol Crystallogr 66:486–501. doi:10.1107/S0907444910007493. 20383002 PMC2852313

[46] Vagin A, Teplyakov A 1997 *MOLREP*: an automated program for molecular replacement. J Appl Crystallogr 30:1022–1025. doi:10.1107/S0021889897006766.

[47] Winn MD, Ballard CC, Cowtan KD, Dodson EJ, Emsley P, Evans PR, Keegan RM, Krissinel EB, Leslie AGW, McCoy A, McNicholas SJ, Murshudov GN, Pannu NS, Potterton EA, Powell HR, Read RJ, Vagin A, Wilson KS 2011 Overview of the CCP4 suite and current developments. Acta Crystallogr D Biol Crystallogr 67:235–242. doi:10.1107/S0907444910045749. 21460441 PMC3069738

[48] Ceccaldi P, Rendon J, Léger C, Toci R, Guigliarelli B, Magalon A, Grimaldi S, Fourmond V 2015 Reductive activation of *E. coli* respiratory nitrate reductase. Biochim Biophys Acta Bioenerg 1847:1055–1063. doi:10.1016/j.bbabio.2015.06.007. 26073890

[49] Fourmond V, Burlat B, Dementin S, Arnoux P, Sabaty M, Boiry S, Guigliarelli B, Bertrand P, Pignol D, Léger C 2008 Major Mo(V) EPR signature of *Rhodobacter sphaeroides* periplasmic nitrate reductase arising from a dead-end species that activates upon reduction. Relation to other molybdoenzymes from the DMSO reductase family. J Phys Chem B 112:15478–15486. doi:10.1021/jp807092y. 19006273

[50] Bard AJ, Faulkner LR 2001 Electrochemical methods: fundamentals and applications. John Wiley & Sons, New York, NY.

[51] Fourmond V 2016 QSoas : a versatile software for data analysis. Anal Chem 88:5050–5052. doi:10.1021/acs.analchem.6b00224. 27096413

[52] Díaz-Moreno I, Díaz-Quintana A, Ubbink M, De La Rosa MA 2005 An NMR-based docking model for the physiological transient complex between cytochrome f and cytochrome c_6_. FEBS Lett 579:2891–2896. doi:10.1016/j.febslet.2005.04.031. 15876432

[53] Shi Z, Zachara JM, Shi L, Wang Z, Moore DA, Kennedy DW, Fredrickson JK 2012 Redox reactions of reduced flavin mononucleotide (FMN), riboflavin (RBF), and anthraquinone-2,6-disulfonate (AQDS) with ferrihydrite and lepidocrocite. Environ Sci Technol 46:11644–11652. doi:10.1021/es301544b. 22985396

[54] Aliverti A, Curti B, Vanoni MA 1999 Identifying and quantitating FAD and FMN in simple and in iron-sulfur-containing flavoproteins, p 9–24. *In* Chapman SK, Reid GA (ed), Flavoprotein protocols, Humana Press, Totowa, NJ. 10.1385/1-59259-266-X:9 10494539

[55] Whitby LG 1953 A new method for preparing flavin-adenine dinucleotide. Biochem J 54:437–442. doi:10.1042/bj0540437. 13058921 PMC1269010

[56] Wood E 1987 Data for Biochemical Research (third edition): by R M C Dawson, D C Elliott, W H Elliott and K M Jones, pp 580. Oxford Science Publications, OUP, Oxford, 1986. Biochem Ed 15:97. doi:10.1016/0307-4412(87)90110-5.

[57] Dixon M 1971 The acceptor specificity of flavins and flavoproteins. III. Flavoproteins Biochim Biophys Acta Bioenerg 226:269–284. doi:10.1016/0005-2728(71)90094-6. 4396857

