# Supplementary material for: How Thermophilic Gram-Positive Organisms Perform Extracellular Electron Transfer: Characterization of the Cell Surface Terminal Reductase OcwA
Source: mBio. 2019 Aug 20;10(4):e01210-19. doi: 10.1128/mBio.01210-19 (PMC6703420; doi:10.1128/mBio.01210-19)
Supplement: TABLE S1 [file mBio.01210-19-st001.docx]

**Table SM1.** Data collection and Refinement Statistics.

| **Data Set** | **OcwA – SAD** | **OcwA – high res** |
| --- | --- | --- |
| space group | *P1* | *P1* |
| wavelength [Å] | 1.728963 | 1.00000 |
| cell constants *a*, *b*, *c* [Å]  α, β, γ [°] | 55.48, 63.16, 84.53  101.81, 99.38, 98.24 | 54.85, 62.97, 84.21 101.54, 99.13, 98.40 |
| resolution limits [Å] | 49.61 – 2.70  (2.83 – 2.70) | 49.06 – 2.20 (2.27 – 2.20) |
| completeness (%) | 89.9 (90.2) | 98.1 (97.3) |
| unique reflections | 26,907 (3,591) | 53,356 (4,603) |
| multiplicity (%) | 3.7 (3.8) | 3.5 (3.4) |
| *R*_merge_ ^a^ | 0.071 (0.35) | 0.075 (1.29) |
| *R*_p.i.m._ | 0.043 (0.21) | 0.047 (0.83) |
| mean I/ σ(I) | 11.7 (3.5) | 11.0 (1.0) |
| CC (1/2) (Karplus and Diederichs, 2012) | 0.996 (0.895) | 0.998 (0.4) |
| refinement statistics | | |
| *R*_cryst_ ^b^ |  | 20.2 |
| *R*_free_ (%) |  | 23.3 |
| non-hydrogen atoms |  | 8280 |
| solvent molecules |  | 1 |
| Cruickshank’s DPI (Cruickshank, 1999) |  | 0.31 |
| r.m.s. deviations from ideal values | | |
| bond lengths (Å) |  | 0.010 |
| bond angles (º) |  | 1.416 |
| protein main chain atoms |  | 59.40 |
| protein all atoms |  | 60.44 |
| solvent |  | 79.99 |
| Wilson plot |  | 58.67 |
| Residues in most favoured regions |  | 97.3 % (937/963) |
| in additional allowed regions |  | 99.8% (961/963) |
| in generously allowed region |  |  |
| ^a^ *R*_merge_ = Σ*_hkl_* [(Σ*_i_* \|*I_i_* - ‹*I*›\|) / Σ*_i_ I_i_*]  ^b^ *R*_cryst_ = Σ*_hkl_* \|\|*F*_obs_\| - \|*F*_calc_\|\| / Σ*_hkl_* \|*F*_obs_\|  *R*_free_ is the cross-validation *R* value for a test set of 5 % of unique reflections  ^c^ Ramachandran statistics as defined by Molprobity (Laskowski et al. 1993) | | |

# References

Cruickshank DWJ (1999) Remarks about protein structure precision. Acta Crystallogr Sect D Biol Crystallogr 55(3):583–601.

Karplus PA, Diederichs K (2012) Linking Crystallographic Model and Data Quality. *Science* 336(6084):1030–1033.

Laskowski RA, MacArthur MW, Moss DS, Thornton JM (1993) PROCHECK: a program to check the stereochemical quality of protein structures. *J Appl Crystallogr* 26(2):283–291.
